# Crocodile blood supplementation protects vascular function in diabetic mice

**DOI:** 10.1186/s43014-021-00066-w

**Published:** 2021-08-03

**Authors:** Chui Yiu Bamboo Chook, Francis M. Chen, Gary Tse, Fung Ping Leung, Wing Tak Wong

**Affiliations:** 1 School of Life Sciences, Faculty of Science, The Chinese University of Hong Kong, Shatin, NT Hong Kong SAR; 2 State Key Laboratory of Agrobiotechnology, The Chinese University of Hong Kong, Hong Kong, China

**Keywords:** Crocodile blood, Diabetes mellitus, Vascular endothelial function, Anti-oxidative, Anti-inflammatory

## Abstract

**Abstract:**

Cardiovascular disease is a major cause of mortality in diabetic patients due to the heightened oxidative stress and pro-inflammatory state in vascular tissues. Effective approaches targeting cardiovascular health for diabetic patients are urgently needed. Crocodile blood, an emerging dietary supplement, was suggested to have anti-oxidative and anti-inflammatory effects in vitro, which have yet to be proven in animal models. This study thereby aimed to evaluate whether crocodile blood can protect vascular function in diabetic mice against oxidation and inflammation. Diabetic *db/db* mice and their counterparts *db/m*^*+*^ mice were treated daily with crocodile blood soluble fraction (CBSF) or vehicle via oral gavage for 4 weeks before their aortae were harvested for endothelium-dependent relaxation (EDR) quantification using wire myograph, which is a well-established functional study for vascular function indication. Organ culture experiments culturing mouse aortae from C57BL/6 J mice with or without IL-1β and CBSF were done to evaluate the direct effect of CBSF on endothelial function. Reactive oxygen species (ROS) levels in mouse aortae were assessed by dihydroethidium (DHE) staining with inflammatory markers in endothelial cells quantified by quantitative polymerase chain reaction (qPCR). CBSF significantly improved deteriorated EDR in *db/db* diabetic mice through both diet supplementation and direct culture, with suppression of ROS level in mouse aortae. CBSF also maintained EDR and reduced ROS levels in mouse aortae against the presence of pro-inflammatory IL-1β. Under the pro-inflammatory state induced by IL-1β, gene expressions of inflammatory cytokines were downregulated, while the protective transcripts UCP2 and SIRT6 were upregulated in endothelial cells. Our study suggests a novel beneficial effect of crocodile blood on vascular function in diabetic mice and that supplementation of diet with crocodile blood may act as a complementary approach to protect against vascular diseases through anti-oxidation and anti-inflammation in diabetic patients.

**Graphical abstract:**

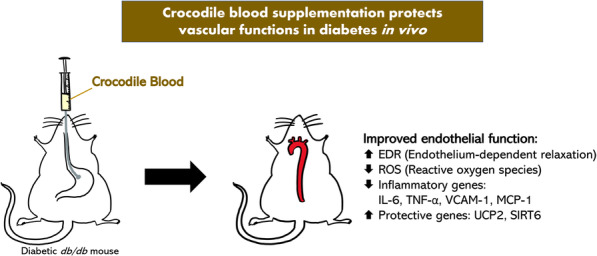

**Supplementary Information:**

The online version contains supplementary material available at 10.1186/s43014-021-00066-w.

## Highlights

- Daily oral crocodile blood supplementation for 5 weeks ameliorated endothelial dysfunction in *db/db* diabetic mice through suppressing the overproduced reactive oxygen species (ROS).
- Direct action of improving endothelium-dependent relaxation (EDR) and lowering ROS level on mouse aortae were demonstrated using ex vivo organ culture experiments on mouse aortae.
- Vasoprotective effects observed against inflammation-induced endothelial dysfunction provides strong evidence and a possible mechanism for the beneficial effects of crocodile blood on vascular health in diabetes.

## Background

One in ten people worldwide suffer from diabetes mellitus (Saeedi et al. 2019), and one in three type 2 diabetic patients has cardiovascular disease (Einarson et al. 2018). While currently used therapeutic strategies for diabetic patients focus mainly on lowering blood glucose levels, diabetes medications only modestly improve cardiovascular disease outcomes, without significantly reducing cardiovascular mortality in diabetic patients (Kelly 2009; Turnbull et al. 2009). Therefore, there is a need for new approaches to improve cardiovascular health in diabetes mellitus patients. In particular, suppression of reactive oxygen species (ROS) and inflammation were suggested to be a mechanism-based therapeutic approach due to its pivotal role in the pathology of diabetic cardiovascular disease (Giacco & Brownlee 2010).

Crocodile blood, marketed as a dietary supplement since 2008 (Chaeychomsri et al. 2013), has been suggested to have a wide range of physiological benefits (Chook et al. 2021), including anti-oxidative (Jangpromma et al. 2018; Lueangsakulthai et al. 2018; Phosri et al. 2014, 2017; Theansungnoen et al. 2014), anti-inflammatory (Jangpromma et al. 2017; Kommanee et al. 2014; Lueangsakulthai et al. 2018; Pata et al. 2011; Phosri et al. 2014, 2017), anti-microbial (Aree et al. 2011; Hao et al. 2012; Kommanee et al. 2012; Merchant et al. 2011; Pata et al. 2007, 2011; Phupiewkham et al. 2018), anti-viral (Kozlowski et al. 2016), anti-tumor (Maijaroen et al. 2018; Maraming et al. 2018; Ou & Ho 2016; Patathananone et al. 2015; Phosri et al. 2018), wound healing enhancing (Jangpromma et al. 2016; Pakdeesuwan et al. 2017) and anti-anemia (Chaeychomsri 2015) effects. Novel functional substances such as Leucrocins (Pata et al. 2011) and crocosin (Preecharram et al. 2010) were identified from crocodile blood. Crocodile blood has thus attracted growing attention and is anticipated to bring new hope to different fields of medications (Smith 2019).

We thereby hypothesized that crocodile blood may have protective effects on vascular endothelial function against diabetic damage through anti-oxidation and anti-inflammation. Using endothelium-dependent relaxation (EDR) as an indicator (Versari et al. 2009), this study examined the effects of crocodile blood on endothelial dysfunction in *db/db* diabetic mice, aiming to evaluate the potential of crocodile blood supplementation on improving vascular health in diabetic patients.

## Materials and methods

### Preparation of crocodile blood soluble fraction

Crocodile blood collected from farmed crocodiles in Thailand was first sterilized at high temperature, with further confirmation on the absence of virus or pathogens warranted. The blood was then freeze-dried, and ground into powder. Upon receival, the crocodile blood powder (HK JEBN Limited, HK) was dissolved in phosphate-buffered saline (PBS). The mixture was then centrifuged at 3900×g for 15 mins and filtered through a 0.22 μm filter to obtain a clear pale-yellowish solution, noted as crocodile blood soluble fraction (CBSF) in this article. All the weight of CBSF in the doses (in vivo experiments) or concentrations (in vitro experiments) refer to the weight of dissolved freeze-dried crocodile blood powder.

### Animal models

Animal experiments were performed on male type 2 diabetic *db/db* mice lacking the gene encoding for leptin receptor from C57BL/KSJ background, the counterpart heterozygote *db/m*^*+*^ mice and C57BL/6 J mice, which were supplied by the Laboratory Animal Service Center (LASEC), the Chinese University of Hong Kong (CUHK), with the approval from the Animal Experimentation Ethics Committee, CUHK (Ref No. 18–243-MIS). Mice were kept in a temperature-controlled holding room (22–24 °C) with a 12-h light/dark cycle. Standard diet and water were provided ad libitum. All *db/db* mice have established blood glucose level over 33.3 mmol/dL before they were randomly divided into 2 groups. *Db/m*^*+*^ mice were also divided into 2 groups without any significant difference in body weight nor blood glucose. At 10 weeks of age, the *db/db* and *db/m*^*+*^ mice were treated with 25 mg/kg body weight/day CBSF for 4 weeks or vehicle (PBS) via oral gavage. Blood was collected into heparinized tubes after anesthetizing the mice before sacrifice.

### Basic parameters

Glucose, total cholesterol, and triglyceride concentrations of the supernatant plasma from centrifuged blood were determined using Glucose LiquiColor®, LiquiColor® Triglycerides and Cholesterol LiquiColor® tests (Stanbio, Boerne, TX, USA) respectively as described in the provided protocols. Blood pressures were measured using the tail-cuff sphygmomanometer (CODA® High Throughput System, Kent Scientific Corporation, Torrington, CT, USA).

### Isometric force measurement

After euthanizing the mice by carbon dioxide (CO_2_) inhalation, thoracic aortae were rapidly removed and immersed in ice-cold oxygenated Krebs-Henseleit solution. Each aorta, with its perivascular adipose tissue removed, was cut into 2-mm segments of aortic rings. Changes in isometric tension of the aortic rings were measured and recorded in the Multi Myograph System (Danish Myo Technology, Aarhus, Denmark) as previously described (Huang et al. 2003). Each aortic ring was mounted to one chamber on the Multi Myograph System using two wires and stretched to an optimal baseline tension of 3 mN. The aortic rings were allowed to equilibrate in the chamber for 60 mins before the start of experiment. 60 mmol/L KCl-containing Krebs solution was added to induce contraction of the aortic rings, which were then rinsed by Krebs solution to restore the baseline tension. Following the contraction induced by 3 μmol/L phenylephrine (Phe), endothelium-dependent relaxation (EDR) was studied along the cumulative addition of acetylcholine (ACh) from 3×10^− 9^ to 1×10^− 5^ mol/L, which stimulates endothelial cells to produce the vasodilator nitric oxide (NO). The aortae were then incubated with L-N^G^-nitro-L-arginine methyl ester (L-NAME) to inhibit NO production by endothelial nitric oxide synthase (eNOS) in the endothelial cells. Endothelium-independent relaxation was then studied in a similar manner as EDR instead using cumulative addition of sodium nitroprusside (SNP) (1×10^− 9^ to 1×10^− 5^ mol/L), which donates NO directly to smooth muscle cells to induce vasodilation. Each experiment was performed on rings prepared from different mice in duplicates.

### Organ culture of mouse aortic rings

Aortic rings were obtained as previously described and incubated with or without CBSF overnight in Dulbecco’s Modified Eagle’s Media (DMEM, Gibco, Gaithersberg, MD, USA) culture media with 1 g/L glucose, 10% fetal bovine serum (FBS, Gibco), 100 IU/mL penicillin and 100 μg/mL streptomycin (Penicillin-Streptomycin, Gibco). Aortic rings from *db/m*^*+*^ and *db/db* mice were divided into 4 groups: *db/m*^*+*^ alone, *db/m*^*+*^ + 0.4 μg/mL CBSF, *db/db* alone, *db/db* + 0.4 μg/mL CBSF. C57BL/6 J mouse aortae were separated into groups of control, 1 pg/mL IL-1β and 1 pg/mL IL-1β + 0.2 μg/mL CBSF. All aortic rings were incubated overnight in a CO_2_ incubator at 37 °C. All experiments on cultured aortic rings were done in duplicates.

### Detection of intracellular reactive oxygen species by dihydroethidium fluorescence

The 2-mm aortae were put into optimal cutting temperature compound (O.C.T.) (Tissue-Tek® O.C.T.™ Compound, Sakura Finetek Europe B.V., Alphen aan den Rijn, Netherland), which were then snap frozen in liquid nitrogen for embedding. The embedded aortae were cut into 5 μm-thick slides for staining with 5 μM dihydroethidium (DHE) (Invitrogen, Waltham, MA, USA) staining solution, which is blue in the cytosol and turns red when oxidized and intercalated with the nucleus. The emission of red light was detected by confocal microscope (TCS SP8 MP, Leica, Wetzlar, Germany). The DHE-emitted light intensity (605 nm) was normalized by the autofluorescence area (488 nm) from each aortic ring. Any DHE detection was done on aortic rings from the same mouse in triplicates.

### Endothelial cell culture

Mouse brain microvascular endothelial cells (mBMECs) (Angio-Proteomie, Boston, MA, USA) were cultured in DMEM (Gibco) with 4.5 g/L glucose, 0.5% FBS (Gibco), 100 IU/mL penicillin and 100 μg/mL streptomycin (Penicillin-Streptomycin, Gibco) at 37 °C in a CO_2_ incubator, divided into groups of control, 1 pg/mL IL-1β and 1 pg/mL IL-1β + 0.2 μg/mL CBSF.

### Cell proliferation assay

Equal number of mBMECs in 100 μL culture medium were seeded into each well of 96-well plate and supplemented with 0.2–51.2 μg/mL of CBSF. After overnight incubation, 50 μL/well Activated-XTT Solution (XTT Cell Proliferation Assay Kit, Manassas, VA, USA) was added, followed by reading the absorbance at 475 nm (specific absorbance) and 660 nm (non-specific absorbance).

### Quantitative polymerase chain reaction (qPCR)

RNA was extracted from homogenized mBMEC samples using TRIzol (Invitrogen) reagent. RNA was converted into cDNA using PrimeScript™ RT Master Mix (TaKaRa, Kyoto, Japan) on Veriti™ 96-Well Thermal Cycler (ThermoFisher Scientific, Waltham, MA, U.S.). Quantitative polymerase chain reaction (qPCR) experiments were performed on the CFX96 Touch™ Real-time PCR Detection System (Bio-Rad Laboratories, Hercules, CA, USA) with QuantiNova SYBR Green PCR kit (Qiagen, Hilden, Germany). Relative expression levels of mRNAs were calculated in relative to *36B4* as the housekeeping gene using the 2^(−Delta Delta C(T)) Method. Primers used for qPCR analysis are available upon request.

### Drugs and solutions

IL-1β was purchased from PeproTech (Rocky Hill, NJ, USA). DHE was purchased from Abcam (Cambridge, UK). Acetylcholine (ACh), L-N^G^-nitro-L-arginine methyl ester (L-NAME), phenylephrine (Phe), and sodium nitroprusside (SNP) were purchased from Sigma-Aldrich Chemical (St Louis, MO, USA) and dissolved in double-distilled water. Krebs-Henseleit solution is composed of (mmol/L): 119 NaCl, 4.7 KCl, 2.5 CaCl_2_, 1 MgCl_2_, 25 NaHCO_3_, 1.2 KH_2_PO_4_, and 11 D-glucose.

### Statistical analysis

Results were expressed as means ± SEM for each group. Statistical significance was determined by two-tailed Student’s *t*-test or one-way ANOVA when appropriate using GraphPad Prism software (Version 8.0, San Diego, CA, USA). A Bonferroni correction was performed for multiple comparisons. *P* < 0.05 was regarded as statistically significant.

## Results

### Safety and toxicity

Prior to receival as powder in our laboratory, potential pathogens such as fungi, bacteria and virus in the crocodile blood samples were eliminated through high-temperature sterilization and lyophilization (Barba et al. 2017; Unger et al. 2009).

A dose-dependent trial was done on C57BL/6 J mice to evaluate the toxicity of crocodile blood (Suppl. Fig. 1). Five groups of mice were treated with different doses of crocodile blood: PBS (vehicle), 65, 130, 190 and 250 mg/kg. All mice showed no observable behavioral changes. The body weight (Suppl. Fig. 1A) and non-fasting blood glucose (Suppl. Fig. 1B) of the mice also showed no significant difference between each group. Cytotoxicity on mBMECs was assessed by XTT assay. No significant difference in cell viability resulted from treatments of concentrations ranged from 0.2 to 51.2 μg/mL CBSF (Suppl. Fig. 1C).

### Basic parameters

**Fig. 1:**
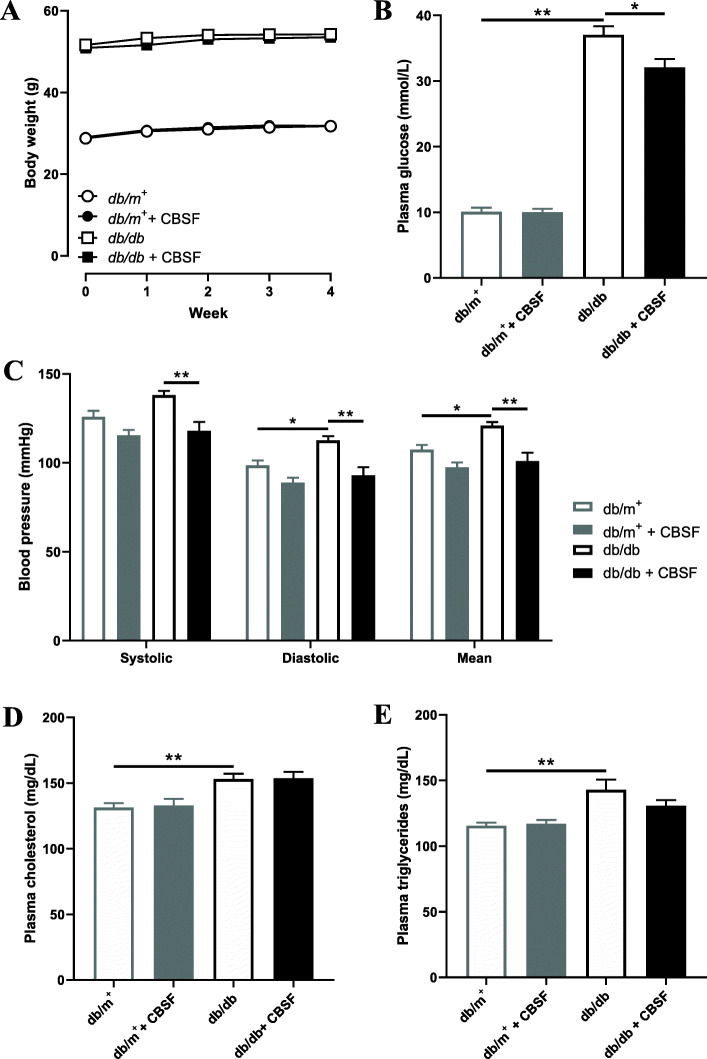
Effects of oral CBSF treatment on the basic parameters of *db/m*^*+*^ and *db/db* mice. Body weight (**a**), plasma glucose level (**b**), systolic blood pressure, diastolic blood pressure, mean arterial pressure (**c**), plasma cholesterol (**d**) and plasma triglycerides (**e**) of *db/m*^*+*^ and *db/db* mice treated or non-treated with CBSF (25 mg/kg/day) via oral gavage for 4 weeks. Data are presented in mean ± SEM; *n* = 8, *p < 0.05 and **p < 0.01. CBSF, crocodile blood soluble fraction

We first evaluated the effects of crocodile blood soluble fraction (CBSF) on the general metabolic health of the mice. Both *db/m*^*+*^ and *db/db* mice treated or non-treated with CBSF showed gradual weight gain over the 4-week oral treatment period with no significant difference between groups (Fig. 1a). Oral CBSF treatment for 4 weeks induced a subtle but significant drop in plasma glucose level in the *db/db* mice, but not in *db/m*^*+*^ mice (Fig. 1b). While the diastolic and mean blood pressures were higher in *db/db* mice than *db/m*^*+*^ mice, CBSF lowered the systolic, diastolic, and mean blood pressures significantly in the *db/db* mice (Fig. 1c). In addition, CBSF had no significant effects on plasma cholesterol (Fig. 1d) nor triglyceride levels (Fig. 1e) in both *db/m*^*+*^ and *db/db* mice.

### In vivo CBSF oral treatment improved endothelium-dependent relaxation (EDR) in *db/db* mouse aortae

**Fig. 2:**
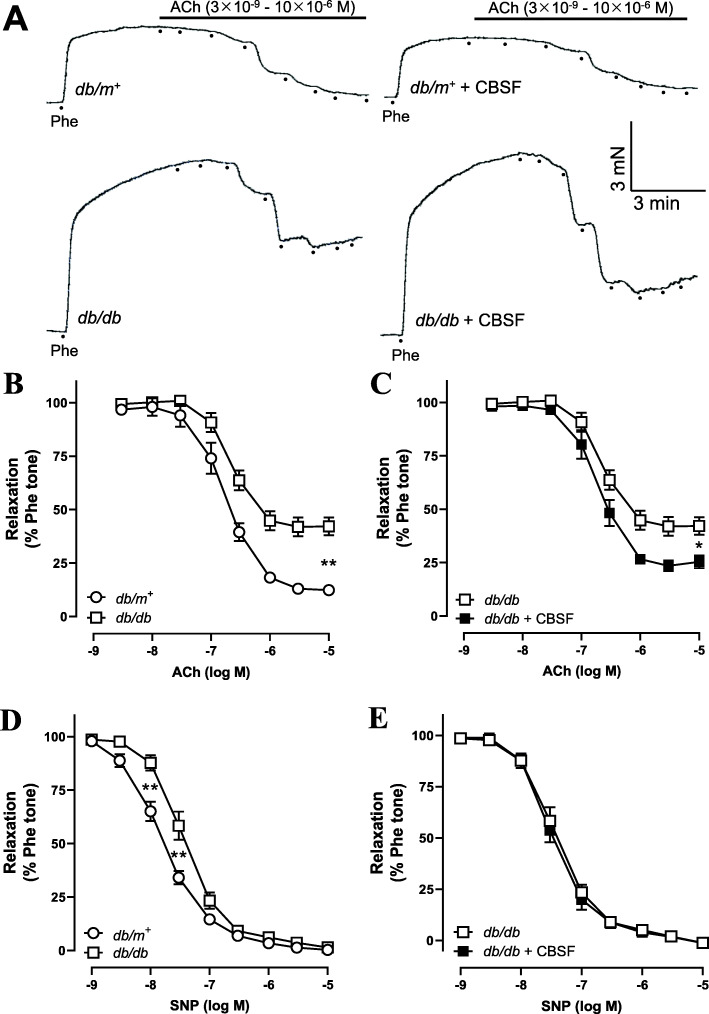
Oral CBSF treatment improved the impaired endothelial-dependent relaxation (EDR) in *db/db* mouse aortae. Representative tracings (**a**) with summarized data (**b-c**) of EDR measured by wire myograph in isolated aortae from *db/m*^*+*^ and *db/db* mice with and without in vivo CBSF treatment (25 mg/kg/day). Summarized data of endothelium-independent relaxation of isolated aortae from *db/m*^*+*^ and *db/db* mice treated or non-treated with CBSF (**d-e**). Data are presented in mean ± SEM; *n* = 8, *p < 0.05 and **p < 0.01 relative to *db/db*. CBSF, crocodile blood soluble fraction; Phe, phenylephrine; ACh, acetylcholine; SNP, sodium nitroprusside

We then examined whether oral CBSF treatment effects endothelial function by wire myograph. The endothelium-dependent relaxation (EDR) induced by the addition of acetylcholine (ACh) was impaired in the *db/db* control mice compared to the *db/m*^*+*^ counterparts (Fig. 2a-b). The 4-week oral CBSF treatment improved the EDR in *db/db* mouse aortae (Fig. 2a & c), yet did not show a significant effect on the *db/m*^*+*^ mouse aortae (Fig. 2a). Following the inhibition of endothelial nitric oxide synthase (eNOS) by L-N^G^-nitro-L-arginine methyl ester (L-NAME), thus inhibiting the ability of endothelial cells to induce vasodilation, the total endothelium-independent relaxation induced by sodium nitroprusside (SNP) was found comparable among the groups, despite a difference in the initial relaxation between *db/m*^*+*^ and *db/db* mice (Fig. 2d-e).

### CBSF directly reversed impaired EDR in ex vivo cultured *db/db* mouse aortae

**Fig. 3:**
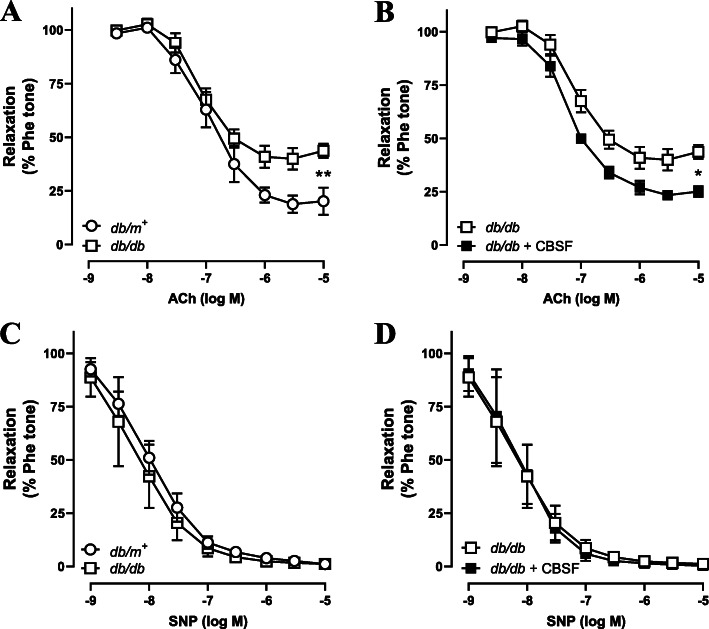
Ex vivo CBSF incubation ameliorated the impaired EDR in *db/db* mouse aortae. EDR measured by wire myograph in non-treated *db/m*^+^ and *db/db* mice (**a**). Effect of ex vivo CBSF incubation on EDR in isolated aortae from non-treated *db/db* mice (**b**). Effect of ex vivo CBSF incubation on endothelium-independent relaxation in isolated aortae from non-treated *db/m*^+^ and *db/db* mice (**c-d**). Data are presented in means ± SEM; *n* = 3 for *db/m+*, *n* = 5 for *db/db*; *p < 0.05, **p < 0.01. CBSF, crocodile blood soluble fraction; Phe, phenylephrine; ACh, acetylcholine; SNP, sodium nitroprusside

Our subsequent experiments investigated whether CBSF has a direct effect on endothelial function by incubating the *db/db* and *db/m*^*+*^ mouse aortae with or without CBSF overnight. While EDR was impaired in the *db/db* group in relative to the *db/m*^*+*^ group (Fig. 3a), CBSF incubation improved the EDR performance in *db/db* mouse aortae (Fig. 3b), without affecting the endothelium-independent relaxation (Fig. 3c-d).

### CBSF reduced the ROS level in *db/db* mouse aortae both in vivo and ex vivo

**Fig. 4:**
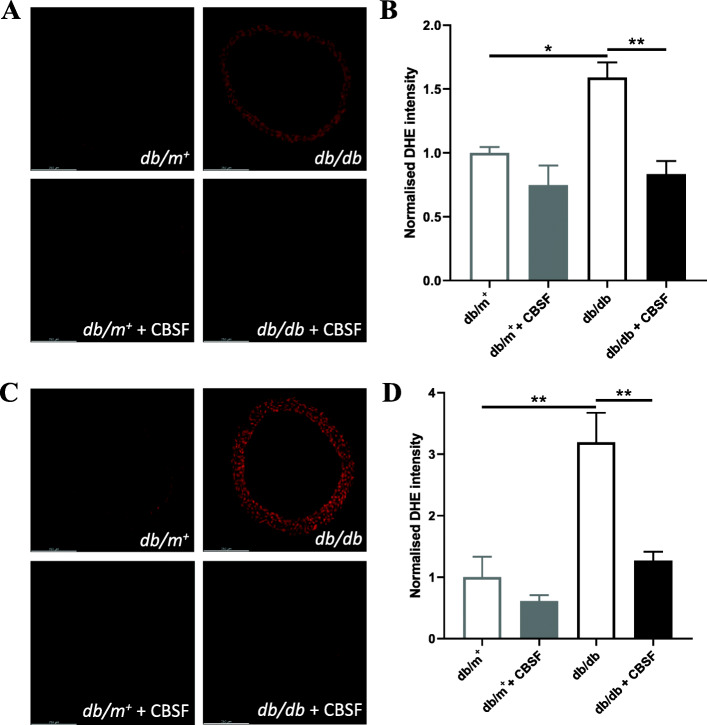
CBSF suppressed the ROS level in *db/db* mouse aortae both in vivo and ex vivo. Representative confocal images (**a**) with summarized data (**b**) of DHE stain intensity in isolated aortic rings from *db/m*^+^ and *db/db* mice with and without in vivo CBSF treatment (25 mg/kg/day). Representative confocal images (**c**) and summarized data (**d**) of DHE stain intensity in isolated aortic rings from non-treated *db/m*^+^ and *db/db* mice with and without ex vivo CBSF incubation (0.4 μg/mL). Data are presented in means ± SEM; *n* = 5; *p < 0.05, **p < 0.01. CBSF, crocodile blood soluble fraction; ROS, reactive oxygen species; DHE, dihydroethidium

Oxidative stress is an important factor leading to endothelial dysfunction in diabetes (Giacco & Brownlee 2010). As reflected by the total dihydroethidium (DHE) intensity, reactive oxygen species (ROS) levels were increased in *db/db* mouse aortic rings as compared to *db/m*^*+*^ control and were lowered by oral CBSF treatment (Fig. 4a-b). In addition, ex vivo CBSF incubation similarly reduced the heightened ROS levels in *db/db* mouse aortic rings (Fig. 4c-d).

### CBSF directly reversed IL-1β-induced EDR impairment in C57BL/6 J mouse aortae

**Fig. 5:**
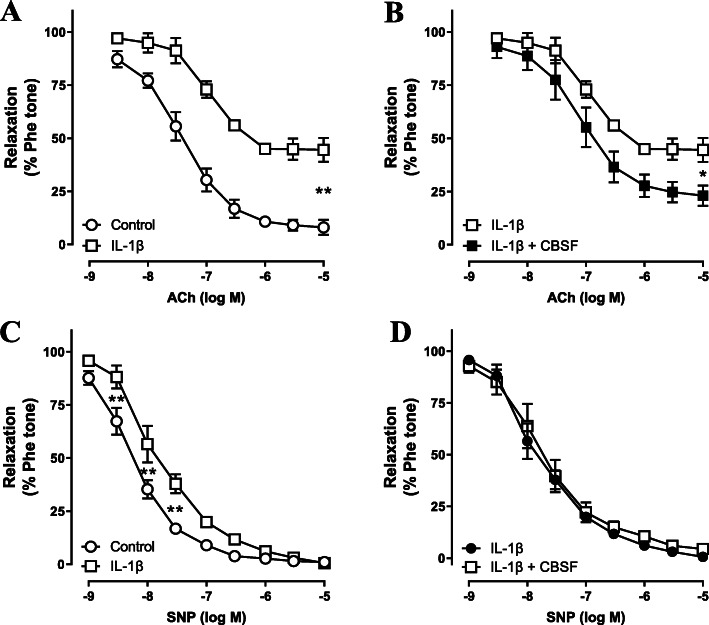
Ex vivo CBSF co-incubation remedied IL-1β-impaired EDR in C57BL/6 J mouse aortae. Effect of IL-1β (1 pg/mL) incubation on EDR of isolated aortae from C57BL/6 J mice (**a**). Effect of CBSF co-incubation on EDR of IL-1β-treated aortae from C57BL/6 J mice (**b**). Effect of IL-1β incubation on endothelium-independent relaxation of isolated aortae from C57BL/6 J mice (**c**). Effect of CBSF co-incubation on endothelium-independent relaxation of IL-1β-treated aortae from C57BL/6 J mice (**d**). Data are presented in means ± SEM; *n* = 5; *p < 0.05, **p < 0.01 compared to IL-1β-treated group. CBSF, crocodile blood soluble fraction; Phe, phenylephrine; ACh, acetylcholine; SNP, sodium nitroprusside

It is well established that inflammation plays an important role in mediating endothelial dysfunction in diabetes (Peiró et al. 2017; Tabit et al. 2010). Using the classical pro-inflammatory cytokine IL-1β, we investigated whether CBSF protects endothelial function against inflammation (Peiró et al. 2016; Sprague & Khalil 2009). Our results showed that EDR of mouse aortae from C57BL/6 J mice was impaired by IL-1β incubation (Fig. 5a), but reversed by co-incubation with CBSF (Fig. 5b). On the other hand, endothelium-independent relaxation either incubated with or without IL-1β was not affected by CBSF (Fig. 5c-d).

### CBSF lowered the ROS level elevated by IL-1β in both normal mouse aortae and mouse brain microvascular endothelial cells (mBMECs)

**Fig. 6:**
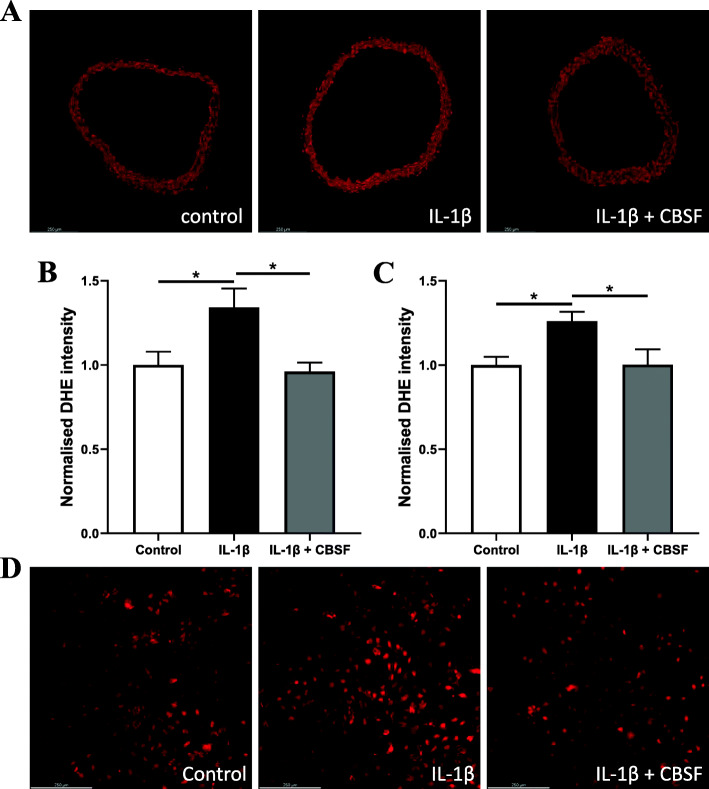
CBSF co-incubation lowered the ROS levels exaggerated by IL-1β in both aortae and endothelial cells. Representative confocal images (**a**) and summarized data (**b**) of DHE stain intensity of C57BL/6 J aortic rings incubated with and without IL-1β (1 pg/mL) and CBSF (0.2 mg/mL). Summarized data (**c**) and representative confocal images (**d**) of DHE stain intensity of mBMECs incubated with and without IL-1β (1 pg/mL) and CBSF (0.2 mg/mL). Data are presented in means ± SEM; *n* = 5; *p < 0.05. CBSF, crocodile blood soluble fraction; ROS, reactive oxygen species; DHE, dihydroethidium

Elevated ROS level was also observed in the IL-1β-incubated mouse aortic rings, but was reduced by CBSF co-incubation (Fig. 6a-b). The high ROS level induced by IL-1β incubation was also lowered by CBSF co-incubation in mBMECs (Fig. 6c-d).

### CBSF downregulated pro-inflammatory cytokines while upregulating protective gene expressions

**Fig. 7:**
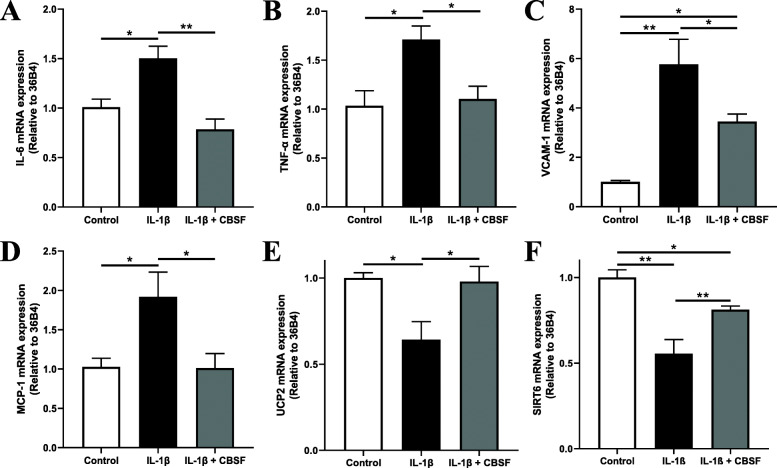
CBSF co-incubation reversed the gene expression levels of pro-inflammatory cytokines, SIRT6 and UCP2 skewed by IL-1β in endothelial cells. Effect of IL-1β (1 pg/mL) and CBSF (0.2 μg/mL) on the mRNA levels of IL-6 (**a**), TNF-α (**b**), VCAM-1 (**c**), MCP-1 (**d**), UCP2 (**e**) and SIRT6 (**f**) in mBMECs. Data are presented in means ± SEM; *n* = 4–6, *p < 0.05, **p < 0.01. CBSF, crocodile blood soluble fraction

We further found that the pro-inflammatory cytokines, IL-6 (Fig. 7a), TNF-α (Fig. 7b), VCAM-1 (Fig. 7c) and MCP-1 (Fig. 7d), upregulated by IL-1β incubation, were reduced by CBSF. In addition, UCP2 (Fig. 7e) and SIRT6 (Fig. 7f) mRNA expression levels downregulated by IL-1β were also restored by CBSF co-incubation.

## Discussion

Endothelial dysfunction is an early manifestation and target for prevention of cardiovascular disease (Versari et al. 2009; Widlansky et al. 2003). Endothelial dysfunction refers to the inability of endothelial cells to maintain vascular homeostasis, which includes the regulation of vasoconstriction, vasodilation, inflammation and oxidative stress (Tabit et al. 2010). Vascular endothelial cells are particularly susceptible to hyperglycemic damage due to limited regulation of glucose transport rate and thus exaggerated glucose influx in high glucose conditions (Kaiser et al. 1993), explaining the especially high prevalence of cardiovascular disease in diabetic patients (Einarson et al. 2018).

In this study, *db/db* mice lacking leptin receptor were used to evaluate the effects of crocodile blood on endothelial functions in type 2 diabetes mellitus. While the 4-week oral CBSF treatment may exert its effects either by direct or systemic influence through regulating blood glucose levels and blood pressure, the EDR improvement by direct CBSF incubation proved a direct protective effect on the endothelial function of CBSF against diabetic damage.

It is well established that upregulation of ROS plays a pivotal role in cardiovascular disease in diabetes (Giacco & Brownlee 2010). Under hyperglycemia, the increased glucose influx leads to increased ROS production. The high oxidative stress not only uncouples eNOS, which further induces overproduction of ROS, but also reduces the bioavailability of NO, which is responsible for vasodilation and anti-inflammation (Giacco & Brownlee 2010; Kim et al. 2006). As a result, a cascade of pro-inflammatory markers including IL-1β, IL-6 and TNF-α were upregulated, eventually leading to endothelial dysfunction, as displayed by EDR impairment (Versari et al. 2009). Our results consistently showed that ROS level increased in the *db/db* mouse aortae was lowered by both in vivo and ex vivo CBSF treatment, indicating that CBSF may possibly protect endothelial function against hyperglycemic damage through ROS suppression.

Nevertheless, exertion of hyperglycemic damage requires pro-inflammatory conditioning (Azcutia et al. 2010; Lafuente et al. 2008; Peiró et al. 2016). It was found that hyperglycemia alone is not sufficient to induce EDR impairment, inflammation and ROS overproduction in blood vessels (Lafuente et al. 2008; Peiró et al. 2016). In the presence of pro-inflammatory IL-1β, however, EDR is impaired, with both ROS production and inflammatory signaling exacerbated by high glucose concentrations (Peiró et al. 2016). The upregulation of VCAM-1 by hyperglycemia in endothelial cells also depends on the presence of IL-1β (Azcutia et al. 2010). Furthermore, IL-1 receptor antagonists were found to inhibit diabetic endothelial dysfunction (Vallejo et al. 2010). Thus, pro-inflammatory cytokines like IL-1β act as a prerequisite for hyperglycemic damage on endothelial function.

In our study, CBSF ameliorated the IL-1β-induced EDR impairment and ROS overproduction in mouse aortae. More specifically, CBSF downregulated the oxidative stress, as well as inflammatory genes in endothelial cells. CBSF also upregulated UCP2 and SIRT6 gene expression in endothelial cells, which were suggested to have a protective role on the endothelial function (Tian et al. 2012; Xu et al. 2017). Thus, our results suggest that CBSF protects endothelial functions against inflammation, which may also be a possible mechanism underlying the protective effect of CBSF on endothelial function in diabetic mice.

Previous studies have shown that single-time oral treatment of crocodile blood lowered inflammatory cytokine production and increased the anti-oxidative enzyme levels in mouse models (Phosri et al. 2017; Phupiewkham et al. 2018). The doses used ranged from 62.5 to 250 mg/kg body weight and caused no observable toxicity to the experimental animals. According to a Thai Congress Report, the protein content of freeze-dried crocodile blood powder is 83.1% (Chaeychomsri et al. 2013). A number of short peptides were isolated from the leucocyte extract and hemoglobin of crocodile blood, and were suggested to possess anti-inflammatory and anti-oxidative effects in vitro (Lueangsakulthai et al. 2018; Phosri et al. 2017; Theansungnoen et al. 2014). However, the content of these peptides in crocodile blood and whether these peptides can also exert the same effects in vivo have not been revealed.

To the best of our knowledge, our study is the first long-term experiment to discover the vasoprotective effects of crocodile blood in vivo. However, given that our study primarily aimed to evaluate the effects of the available crocodile blood supplement on vascular function in *db/db* mouse model, our results have two major limitations. First, detailed molecular mechanisms regarding the effects observed were not identified. Second, the responsible active components in crocodile blood require further investigations. A number of novel peptides identified in crocodile blood were suggested to possess anti-oxidative properties in vitro (Pata et al. 2011; Phosri et al. 2017; Theansungnoen et al. 2014), not to mention the many yet to be discovered. Whether these anti-oxidative peptides contribute to the in vivo vasoprotective effects described in this article is anticipated to be verified in future studies. It is also very likely that multiple active components collaboratively contribute to the described effects through different molecular pathways.

In addition to the previously suggested anti-inflammatory, anti-oxidative, anti-microbial, anti-viral, anti-tumor, anti-anemia, and wound healing enhancing effects, our data indicated that crocodile blood may also protect vascular functions in diabetic patients. Although how the active substances in crocodile blood are digested, absorbed, and processed in the body requires further investigations, this study highlights the presence of vasoactive substances in crocodile blood, and acts as a first step in developing a novel vasoprotective medication or supplementation for diabetic patients. In view of the recent increased incidence of zoonotic diseases in humans such as SARS-CoV-2 and bird flu, further characterization of crocodile blood is needed to confirm the absence of virus and any possible pathogens.

## Conclusion

Our results indicated that CBSF improved vascular endothelial function both in vivo and ex vivo in *db/db* diabetic mice, possibly through ROS suppression. The positive effects also applied to IL-1β-induced inflammation model, with downregulation of pro-inflammatory genes and upregulation of beneficial genes, which may explain how CBSF protects endothelial health in diabetic condition. Although verification of the exact mechanistic pathways requires further study, our study provides insights to the further identification of vasoprotective substances in crocodile blood, which can potentially be developed into a novel medicinal approach for diabetic patients targeting cardiovascular diseases.

## Supplementary Information

**Additional file 1: Supplementary Figure 1**. Crocodile blood does not cause toxicity to C57BL/6 J mice and mBMECs. Body weight (A), and non-fasting blood glucose level (B) of C57BL/6 J mice treated with different doses of crocodile blood via oral gavage for 5 weeks (*n* = 5). Effects of CBSF on the cell viability of mBMECs assessed by XTT Assay (*n* = 8) (C). Data are represented in means ± SEM. CBSF, crocodile blood soluble fraction; mBMECs, mouse brain microvascular endothelial cells.

## Data Availability

The data used to support the findings of this study are included in the article.

## Abbreviations

ACh: Acetylcholine
CBSF: Crocodile blood soluble fraction
DHE: Dihydroethidium
EDR: Endothelium-dependent relaxation
eNOS: Endothelial nitric oxide synthase
L-NAME: L-N^G^-nitro-L-arginine methyl ester
mBMECs: Mouse brain microvascular endothelial cells
NO: Nitric oxide
PBS: Phosphate-buffered saline
Phe: Phenylephrine
qPCR: Quantitative polymerase chain reaction
ROS: Reactive oxygen species
SNP: Sodium nitroprusside
