# Supplementary material for: Crocodile blood supplementation protects vascular function in diabetic mice
Source: Food Prod Process Nutr. 2021 Aug 3;3(1):19. doi: 10.1186/s43014-021-00066-w (PMC8328534; doi:10.1186/s43014-021-00066-w)
Supplement: Supplementary file 1 — Additional file 1: Supplementary Figure 1. Crocodile blood does not cause toxicity to C57BL/6 J mice and mBMECs. Body weight (A), and non-fasting blood glucose level (B) of C57BL/6 J mice treated with different doses of crocodile blood via oral gavage for 5 weeks (n = 5). Effects of CBSF on the cell viability of mBMECs assessed by XTT Assay (n = 8) (C). Data are represented in means ± SEM. CBSF, crocodile blood soluble fraction; mBMECs, mouse brain microvascular endothelial cells. [file 43014_2021_66_MOESM1_ESM.pptx]

## Slide 1
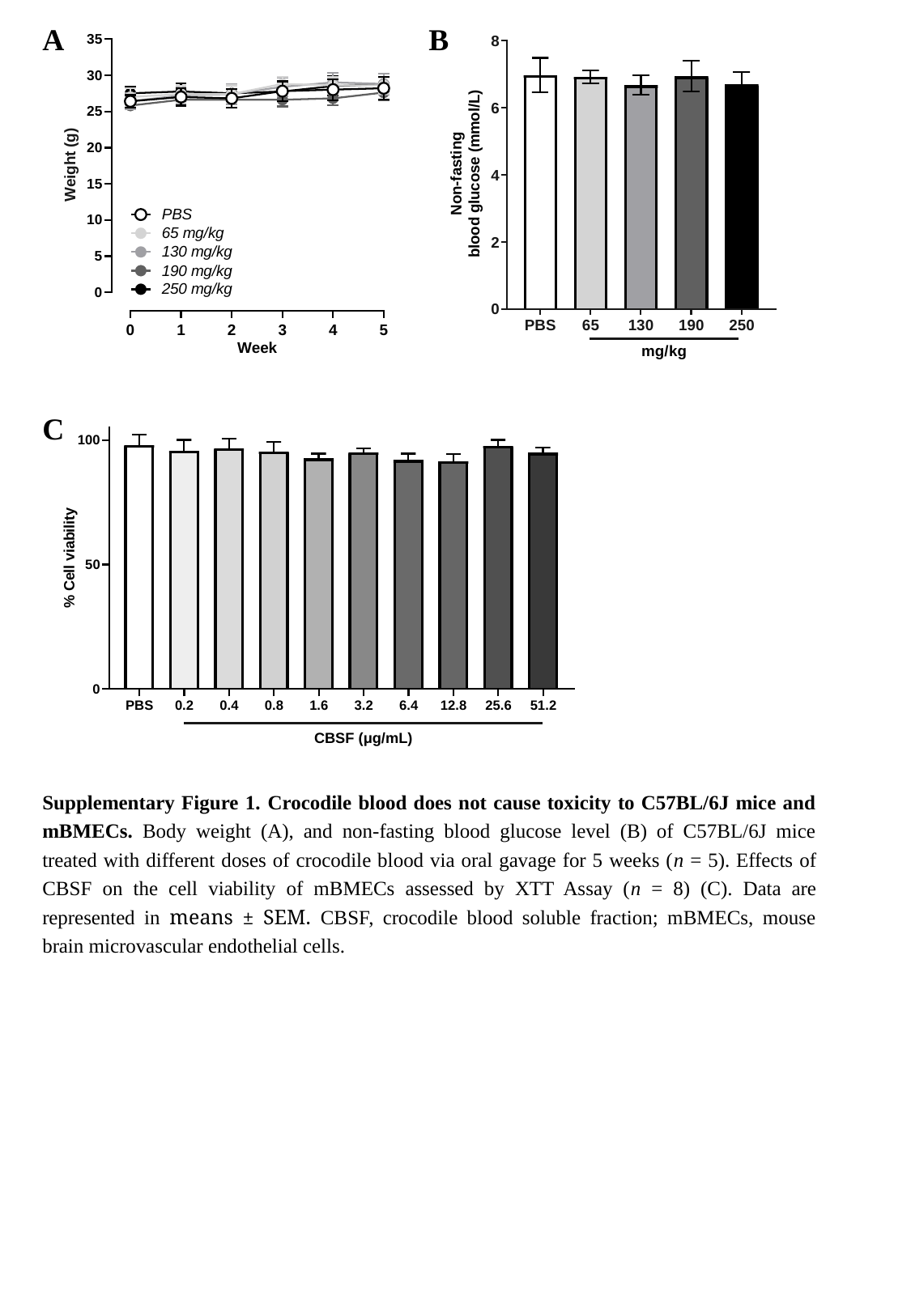

A
B
C
Supplementary Figure 1. Crocodile blood does not cause toxicity to C57BL/6J mice and mBMECs. Body weight (A), and non-fasting blood glucose level (B) of C57BL/6J mice treated with different doses of crocodile blood via oral gavage for 5 weeks (n = 5). Effects of CBSF on the cell viability of mBMECs assessed by XTT Assay (n = 8) (C). Data are represented in means ± SEM. CBSF, crocodile blood soluble fraction; mBMECs, mouse brain microvascular endothelial cells.
